# Polyphyllin II induced apoptosis of NSCLC cells by inhibiting autophagy through the mTOR pathway

**DOI:** 10.1080/13880209.2022.2120021

**Published:** 2022-09-14

**Authors:** Yuhan Jiao, Ming Xin, Juanjuan Xu, Xindong Xiang, Xuan Li, Jingjing Jiang, Xiuqin Jia

**Affiliations:** The Key Laboratory of Molecular Pharmacology, Liaocheng People’s Hospital, Liaocheng, China

**Keywords:** *Rhizoma Paridis*, autophagy inhibition, chloroquine phosphates, 3-methyladenine

## Abstract

**Context:**

Polyphyllin II (PPII) is a steroidal saponin isolated from *Rhizoma Paridis*. It exhibits significant antitumor activity such as anti-proliferation and pro-apoptosis in lung cancer.

**Objective:**

To explore whether PPII induce autophagy and the relationship between autophagy and apoptosis in non-small cell lung cancer (NSCLC) cells.

**Materials and methods:**

The effects of PPII (0, 1, 5, and 10 μM) were elucidated by CCK8 assay, colony formation test, TUNEL staining, MDC method, and mRFP-GFP-LC3 lentivirus transfection in A549 and H1299 cells for 24 h. DMSO-treated cells were selected as control. The protein expression of autophagy (LC3-II, p62), apoptosis (Bcl-2, Bax, caspase-3) and p-mTOR was detected by Western blotting. We explored the relationship between autophagy and apoptosis by autophagy inhibitor CQ (10 μM) and 3-MA (5 mM).

**Results:**

PPII (0, 1, 5, and 10 μM) inhibited the proliferation and induced apoptosis. The IC_50_ values of A549 and H1299 cells were 8.26 ± 0.03 and 2.86 ± 0.83 μM. We found that PPII could induce autophagy. PPII promoted the formation of autophagosome, increased the expression of LC3-II/LC3-I (*p* < 0.05), while decreased p62 and p-mTOR (*p* < 0.05). Additionally, the co-treatment with autophagy inhibitors promoted the protein expression of c-caspase-3 and rate of Bax/Bcl-2 (*p* < 0.05), compared with PPII-only treatment group. Therefore, our results indicated that PPII-induced autophagy may be a mechanism to promote cell survival, although it can also induce apoptosis.

**Conclusions:**

PPII-induced apoptosis exerts its anticancer activity by inhibiting autophagy, which will hopefully provide a prospective compound for NSCLC treatment.

## Introduction

Lung cancer is the leading cause of cancer-related death worldwide (DeSantis et al. 2019; Pal Singh et al. 2020), and non-small cell lung cancer (NSCLC) is the most common type accounting for about 85% of all lung cancer cases. Among NSCLC patients, nearly 70% are the advanced stage or metastatic stage, and the 5-year survival rate is less than 5%. Surgery, chemotherapy, radiation therapy and targeted therapy are the current therapies for NSCLC patients. Platinum-based chemotherapy such as cisplatin plays a vital role in the treatment of advanced or metastatic lung cancer (Ocana et al. 2010; Miller et al. 2019). However, it is usually limited by dose-related toxicity, which leads to the decrease of clinical application effect. Therefore, it is imperative to research and develop the new drugs such as phytochemicals for the treatment of NSCLC.

Apoptosis and autophagy are cell physiological processes mediated by different regulatory molecules (Lockshin and Zakeri 2004; Maiuri et al. 2007; Eisenberg-Lerner et al. 2009; Young et al. 2013). Apoptosis and autophagy have an interactive effect, and this interaction must be regulated by common signalling pathways and proteins. Therefore, the relationship between apoptosis and autophagy is complicated (Green 2002). Autophagy can promote cell survival or death under various cell conditions, but the mechanism is not clear. In recent years, the role of autophagy in cancer treatment has attracted widespread attention (Davies et al. 2003; Liu et al. 2012, 2017). Among them, the key autophagy regulator mTOR (mammalian rapamycin target, mTOR) serves as a central checkpoint for the negative regulation of autophagy and participates in the occurrence and development of various tumours. Most antitumor drugs stimulate autophagy by inhibiting the mTOR pathway, and induce the apoptosis of tumour cells (Kocaturk et al. 2019). Therefore, more evidence indicates that a series of natural products of mTOR-mediated autophagy have possible antitumor effects and favourable prospects in clinical application. Considering the potential dual function of autophagy regulated by the mTOR pathway in tumour survival, we need to further clarify the precise role of autophagy in different tumour types and explore the relationship between autophagy and apoptosis (Xie et al. 2021).

Polyphyllin II (PPII) is a steroidal saponin extracted from the traditional Chinese medicine *Rhizoma Paridis* [*Paris polyphylla* L. (Liliaceae)] (Jing et al. 2017), which has strong physiological and pharmacological effects, such as anti-inflammatory, analgesic, hemostasis, antibacterial and immune regulation, especially antitumor effects (Zhu et al. 2011; Negi et al. 2014; Chen et al. 2016; Pang et al. 2020). According to reports, PPII has significant anti-proliferation and pro-apoptosis in cancer cells such as liver, gliomas ovarian, and colorectal cancer cells (Chen et al. 2019; Niu et al. 2020; Zhao et al. 2020; Callens et al. 2021; Morris and Tjandra 2021). However, there is no relevant research on the antitumor activity of PPII in NSCLC cells and its underlying mechanism, and whether it is related to autophagy remains to be explored and clearly defined. This study investigates the antitumor activity of PPII on NSCLC cells lines and whether it induces autophagy by regulating mTOR pathways. We clarify the relationship between autophagy and apoptosis, and provide experimental evidence for further revealing the molecular mechanisms and exploring its potential therapeutic value in NSCLC.

## Materials and methods

### Materials and reagents

Human NSCLC cell lines A549 and H1299 were acquired from Nanjing Kebai Biological Technology Co., Ltd. (Nanjing, China). PPII was obtained from Dalian Meilun Biotechnology Co., Ltd. (Dalian, China). RPMI-1640 medium, phosphate-buffered saline (PBS), penicillin/streptomycin, dimethyl sulfoxide (DMSO), trypsin-EDTA digestion solution, puromycin, DAPI solution (C0065) were purchased from Solarbio (Beijing, China). Foetal bovine serum (AB-FBS0500) was purchased from ABW (Shanghai, China). The CCK8 kit (c0039), one step TUNEL apoptosis assay kit (C1088) was obtained from Shanghai Beyotime Biotechnology Co., Ltd. (Shanghai, China). Chloroquine phosphates (CQ), 3-methyladenine (3-MA), autophagy staining detection kit (G0170) were purchased from Solarbio (Beijing, China). Anti-bodies against Bcl-2 (12789-1-AP), Bax (50599-2-Ig), caspase-3(19677-1-AP), p62 (66184-1-Ig), LC3 (14600-1-AP), mTOR (20657-1-AP) were purchased from Proteintech (Chicago, IL). β-tubulin (bsm-33034) were obtained from Bioss (Beijing, China). Alexa Fluor 488 Goat anti-Rabbit IgG (H + L) (ab150077) and Alexa Fluor 594 Goat anti-Mouse IgG (H + L) (ab150116) were obtained from Abcam (USA). MAP1LC3B was obtained from Shanghai Jikai Gene Technology Co., Ltd. (Shanghai, China).

### Cells and cell culture

A549 and H1299 cells were cultured in RPMI-1640 medium containing 10% foetal bovine serum in an incubator at 37 °C and 5% CO_2_.

### Preparation of PPII

PPII was dissolved in dimethyl sulfoxide (DMSO) at a stock solution of 50 mM and stored at −20 °C. We prepared the working concentration by diluting the stock solution with culture medium.

### CCK8 assay

The A549 and H1299 cells were seeded in a 96-well plate with 6 × 10^3^ cells per well, treated with five different concentration gradients of PPII for 24 h and then incubated with 10% CCK8 for 2 h. Microplate readers were used to measure the OD value at 570 nm. The cells were pre-treated with CQ (10 μM) or 3-MA (5 mM) for 1 h, with or without PPII (10 μM) for 24 h. Then, cells were incubated with CCK8 for 2 h. The OD value was measured by a microplate reader to calculate IC_50_.

### Colony formation assay

A549 and H1299 cells were inoculated in a 6-well plate at 400–600 cells/well during logarithmic growth, and incubated with various concentrations of PPII after 24 h, except for the control group. After 10–14 days of incubation, we fixed the cells with 4% paraformaldehyde for 1 h, then washed twice and stained with 0.1% crystal violet for 20 min. The number of clones was then counted in each group. Each experiment was performed in triplicate.

### Apoptosis assay

Apoptosis induction was evaluated by TUNEL staining. A549 and H1299 cells were treated with different concentrations of PPII, washed twice with PBS after 24 h, and fixed in 4% (w/v) paraformaldehyde at room temperature for 1 h. Then, the cells were incubated with 0.3% Triton X-100 at room temperature for 5 min and washed twice. Finally, 50 μL TUNEL working solution was added to the wells and incubated at 37 °C for 1 h in the dark. The stained cells were examined with a fluorescence microscope.

### MDC Analysis

MDC is a fluorescent pigment, which is an eosinophilic staining agent. It is usually used to detect the formation of autophagy. After counting the cells, the cells were used to seed 24-well plates with 5 × 10^5^/well. After 24 h of PPII administration with different concentrations, the cells were washed twice with PBS, and 10% MDC working solution (10 μL MDC stain + 90 μL 10 × wash buffer) was prepared 100 μL each well and mixed gently. The cells were stained with MDC working solution at room temperature for 60 min. The fluorescence images were collected and observed by a fluorescence microscope after blocking with anti-fluorescence quencher.

### Analysis of autophagic flux

A549 and H1299 cells were transfected with tandem mRFP-GFP-LC3 lentivirus according to the manufacturer’s instructions. The transfected cells were treated with 0, 1, 5, and 10 μM of PPII for 24 h. Expression images of GFP and mRFP were acquired by a confocal laser scanning microscopy. The changes in autophagic flux were evaluated by the fluorescence of different colours labelled with GFP/mRFP at different concentrations.

### Western blot analysis

A549 and H1299 cells treated with PPII at different concentrations (0, 1, 5, and 10 μM) were collected, washed with PBS and lysed with PMSF, cold RIPA buffer containing protease inhibitors and phosphatase inhibitors for 30 min, and the proteins were collected. We used the BCA kit to determine the protein concentration according to the operating instructions, load the same amount (15 μg/well) of the protein sample into each channel of the SDS-PAGE concentrated gel concentrated gel, and separate it with the SDS-PAGE separating gel, and transfer the protein samples to the PVDF membrane. The membrane was blocked with 5% skim milk for 1 h, and then incubated with the primary antibody overnight. Then washing three times, it was incubated with the secondary antibody for 1 h. After washing three times for 10 min each time, the proteins were detected by ECL western blot detection reagent.

### Statistical analysis

All data were expressed as means ± SD of at least three independent experiments. Differences of independent samples were compared by Student’s *t*-test, while one-way ANOVA was used to analyse the differences between groups. All comparisons with *p* < 0.05 were considered statistically significant.

## Results

### PPII inhibited the proliferation of A549 and H1299 cells

**Figure 1.**
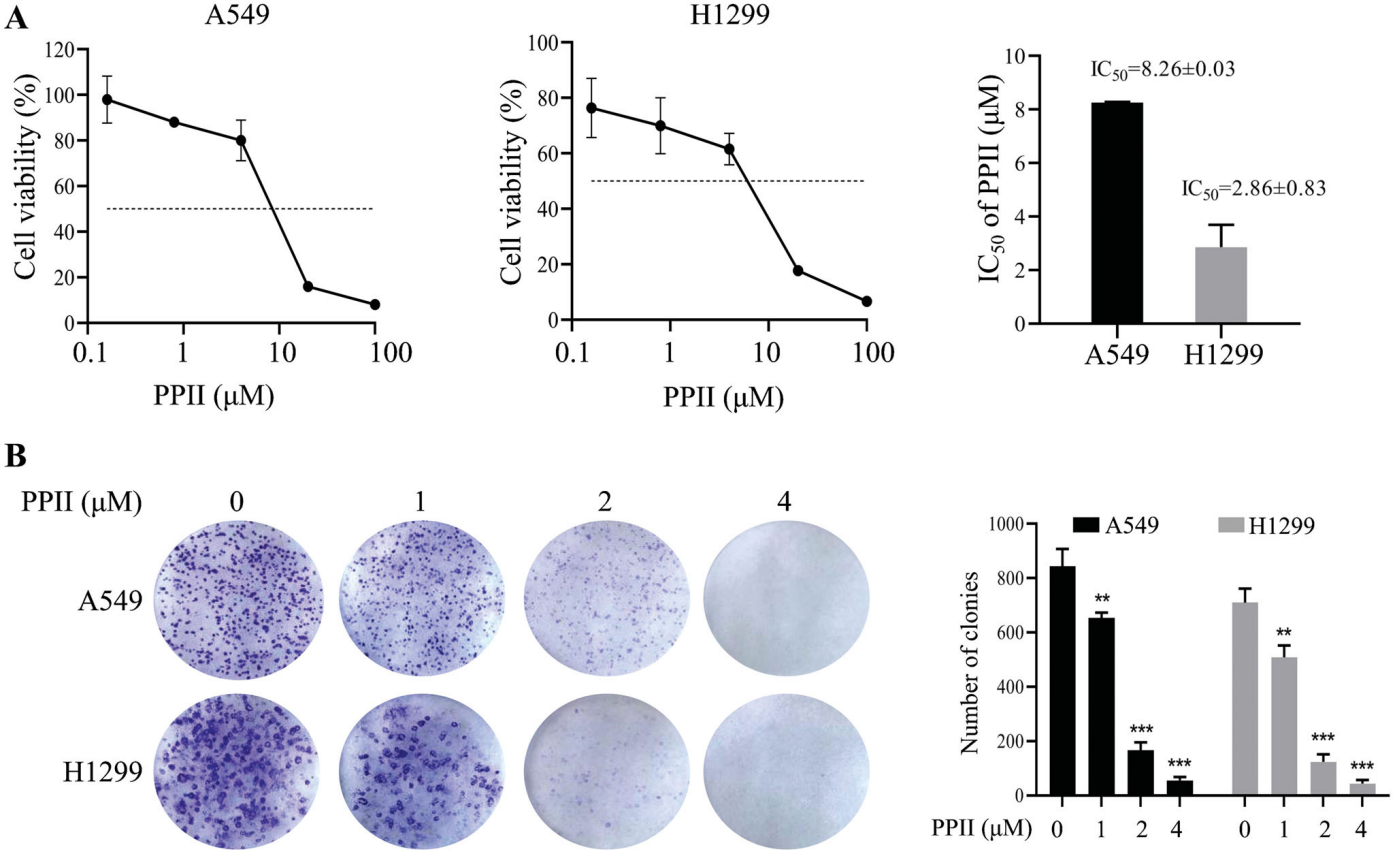
PPII inhibited cell growth in A549 and H1299. (A) Cell viability and IC_50_ value were determined by CCK8 assay after PPII treatment for 24 h. (B) The PPII-treated cells were grown in a 6-well plate for 10–14 days, and the number of cell colonies in each colony was calculated. The data are the mean ± *SD* of independent experiments in three groups. **p < 0.01, ***p < 0.001, compared with the controls.

To examine the anti-proliferative effect of PPII, A549 and H1299 cells were treated with PPII at concentrations of 0, 1, 5, and 10 μM. After 24 h, the cell viability was measured by the CCK8 kit. The data showed that PPII significantly reduced the viability of A549 and H1299 cells when compared with untreated control cells (Figure 1(A)). After PPII treatment for 24 h, the 50% inhibitory concentration values (IC_50_) of A549 and H1299 cells were 8.26 ± 0.03 and 2.86 ± 0.83 μM, respectively. In addition, the cell viability was not significantly different between A549 and H1299 (Figure 1(A)). These results indicated that PPII has a strong anti-proliferative effect on A549 and H1299 cells *in vitro*.

To further study the inhibitory effect of different concentrations of PPII on the growth of A549 and H1299 cells, a clone formation test was carried out. The anti-proliferation ability was detected by counting the clone formation rate. The results showed that the colony-forming ability of A549 and H1299 cells was significantly reduced when treated with PPII (2-4 μM), as shown in Figure 1(B). These results showed that PPII could effectively inhibit the activity of A549 and H1299 cells.

### PPII induced apoptosis of A549 and H1299 cells

**Figure 2.**
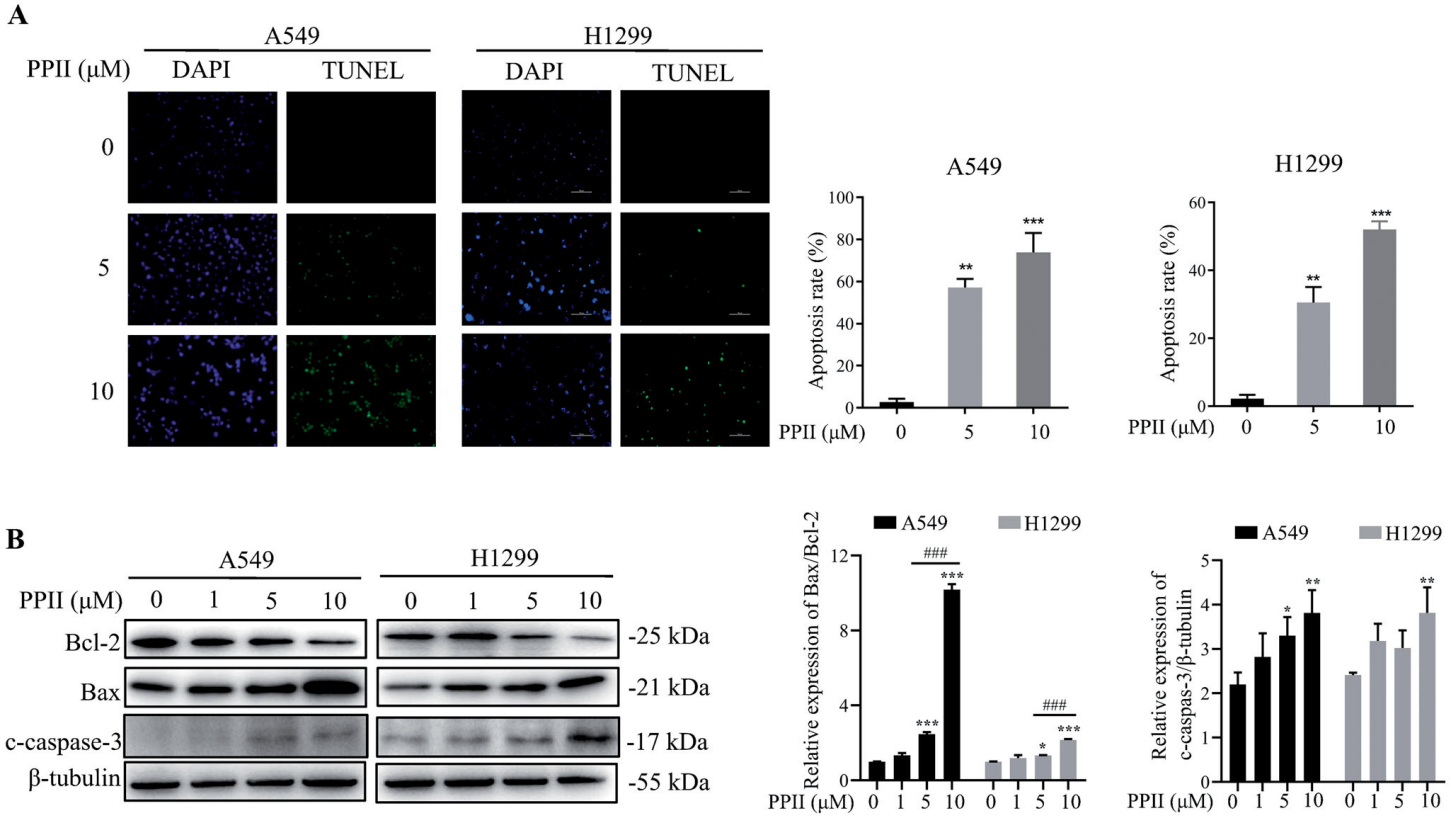
PPII induced apoptosis of A549 and H1299 cells. (A) A549 and H1299 cells were treated with PPII at concentrations of 0, 1, 5, and 10 μM, and then apoptosis was assessed by TUNEL staining. (B) The cells were exposed to PPII for 24 h, and the expression of apoptosis regulatory proteins Bax/Bcl-2, and c-caspase-3 were estimated by western blot analysis. β-Tubulin was used as a loading control. The corresponding expression levels are shown as bar graphs. Data are the mean ± *SD* of three independent experiments. *p < 0.05, **p < 0.01, ***p < 0.001, compared with the controls. ^###^p<0.001, versus PPII (5μM).



TUNEL staining was used to determine whether different concentrations of PPII treatment resulted in apoptosis. After the cells treated with PPII (0, 5, and 10 μM) for 24 h, TUNEL staining showed that A549 and H1299 cells exhibited apoptosis characteristics (Figure 2(A)). The fluorescence intensity of both cells was significantly enhanced with the increasing concentration of PPII. Bcl-2 and Bax proteins are located upstream of mitochondria, which are important regulatory factors of mitochondrial membrane permeability (Morris and Tjandra 2021), and mediate cell survival or death (Callens et al. 2021). Therefore, many studies have shown that the ratio of Bax/Bcl-2 is the key to determining the survival of cells (Walensky 2006). While caspase, a family of cysteine proteases, is the main apoptosis regulator and closely related to the apoptosis signalling pathway. Among them, caspase-3 is a frequently activated death protease that catalyses the specific cleavage of many key cellular proteins (Yadav et al. 2021; Eskandari and Eaves 2022). We then examined the effect of PPII on changes of Bcl-2 and Bax by western blotting. Our results showed that with the increase of PPII concentration, the expression of anti-apoptotic protein Bcl-2 was decreased, while the expression of pro-apoptotic protein Bax was increased, so the ratio of Bax/Bcl-2 increased. In addition, the protein expression of c-caspase-3 decreased (Figure 2(B)). Therefore, we concluded that PPII induced apoptosis in A549 and H1299 cells.

### PPII induced autophagy in A549 and H1299 cells

**Figure 3.**
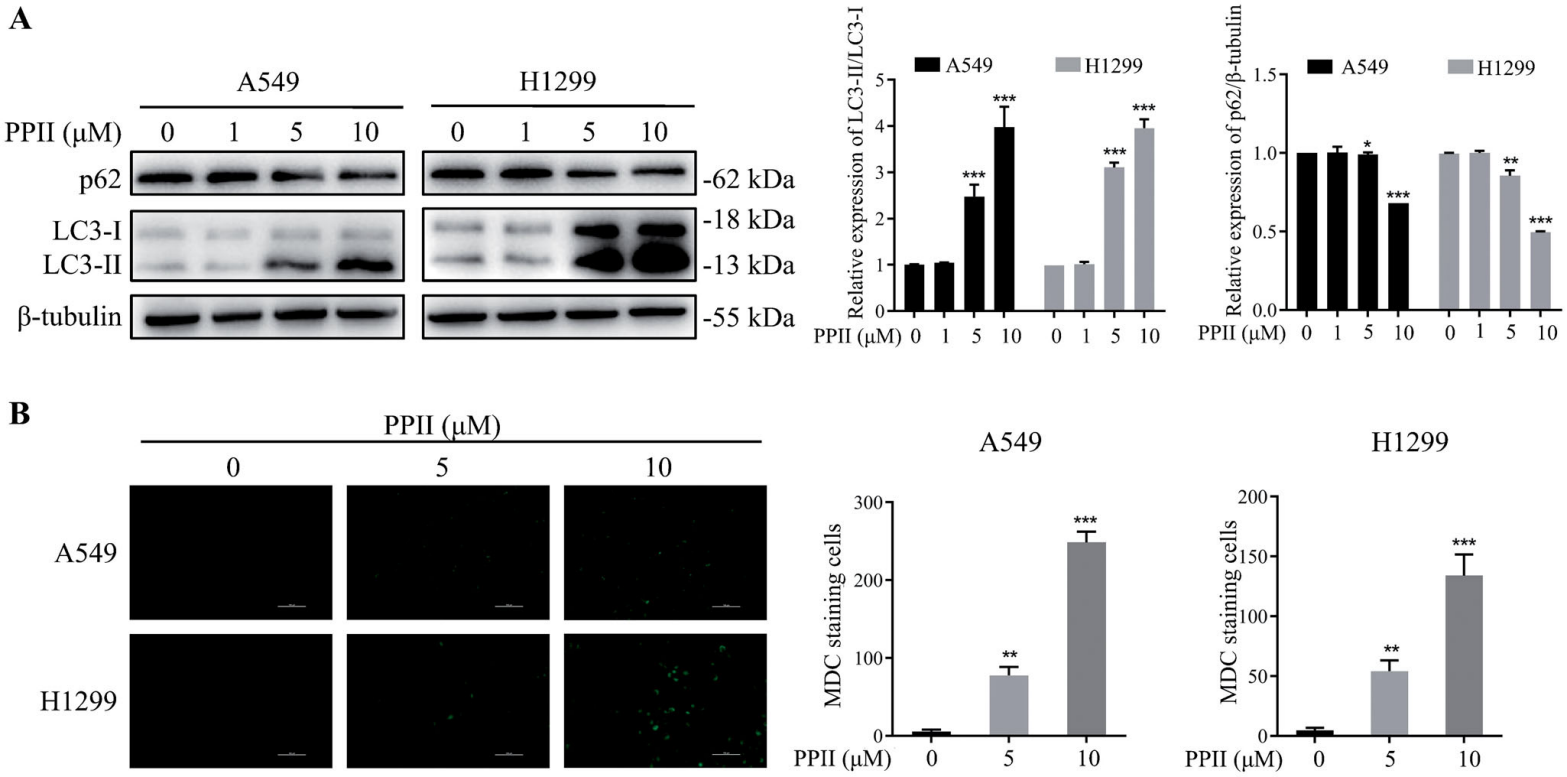
PPII induced autophagy in A549 and H1299 cells. (A) A549 and H1299 cells were treated with PPII at concentrations of 0, 1, 5, and 10 μM for 24 h, and the PPII-induced autophagy was detected by MDC staining. (B) Cells were incubated with PPII (0, 1, 5, 10 μM) for 24 h, and the expression levels of LC3-II/LC3-I, and p62 were examined by western blotting. The corresponding expression levels are shown as bar graphs. Data are mean ± *SD*, representative of three independent experiments. *p < 0.05, **p < 0.01, ***p < 0.001, compared with the controls.

The active ingredients extracted from natural products can cause cell death in various pathways, as well as induce autophagy (Zhang and Liu 2021). Therefore, we investigated whether PPII induced autophagy in human lung cancer cells. The expression of autophagy marker LC3-II/LC3-I and cargo receptor p62 was analysed by western blotting. Our data showed that the conversion rate of LC3-I to LC3-II increased significantly, and the protein expression of p62 was reduced (Figure 3(A)) with the increase of PPII concentration. Next, we observed the MDC staining under a fluorescence microscope. As shown in Figure 3(B), when cells were incubated with PPII (0, 5, 10 μM) for 24 h, we found that the green fluorescence in the cells was enhanced by MDC staining, indicating that PPII promoted the increase of autophagosomes.

### PPII induced autophagic flux in A549 and H1299 cells

**Figure 4.**
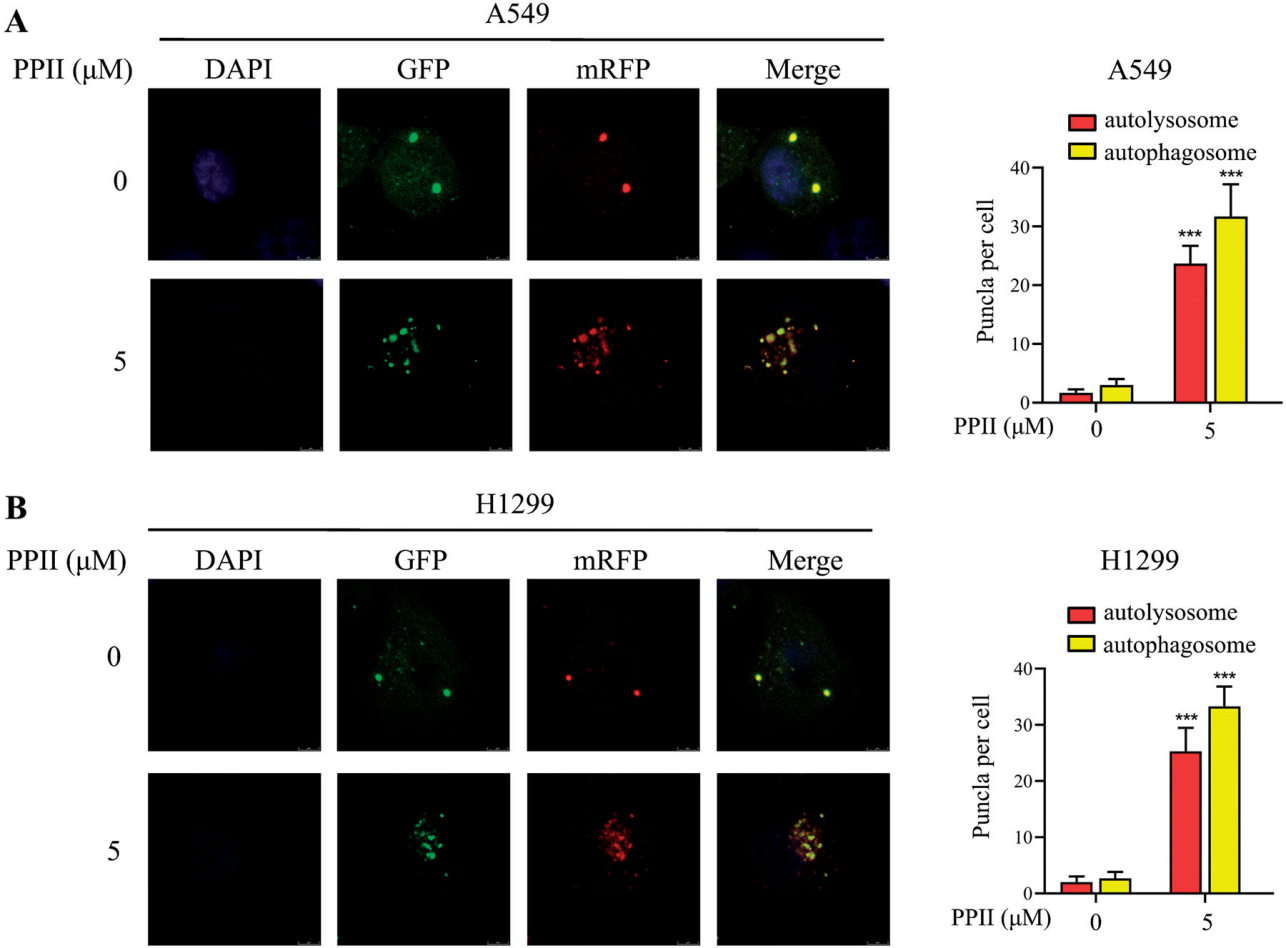
PPII induced autophagic flux in A549 and H1299 cells. (A, B) Cells infected with lentivirus were treated with 5 μM PPII for 24 h, and fluorescence images were observed by a confocal laser scanning microscopy. Red fluorescence represents autolysosome, yellow fluorescence represents autophagosome. Data are mean ± *SD*, representative of three independent experiments. ***p < 0.001, compared with the controls.

To elucidate the effect of PPII on autophagy, we transfected the A549 and H1299 cells with tandem mRFP-GFP-LC3 lentivirus, and then the LC3 protein carried mRFP red fluorescence and GFP green fluorescence. When a large number of epitopes accumulate on the autophagosome membrane, they will appear yellow fluorescence. In the early stages of autophagy, the infected cells produced GFP and mRFP signals. The GFP protein is acid-sensitive and rapidly quenched upon fusion of autophagosomes and lysosomes. In contrast, mRFP is relatively stable in the acidic environment of autophagosomes. Before PPII treatment, only weak GFP and mRFP signals were found in the cytoplasm which represents diffuse LC3 protein. After treatment with PPII for 24 h, the accumulation of mRFP red dots, and yellow dots in the combined graph increased, and the yellow dots were more than the red dots, indicating that autophagy was induced, autophagy flux was increased (Figure 4(A,B)). Therefore, our results showed that PPII promoted an increase in the number of autophagosomes and autophagolysosomes, which confirmed that PPII induced autophagic flux in A549 and H1299 cells.

### PPII induced autophagy by inhibiting the mTOR pathway in A549 and H1299 cells

**Figure 5.**
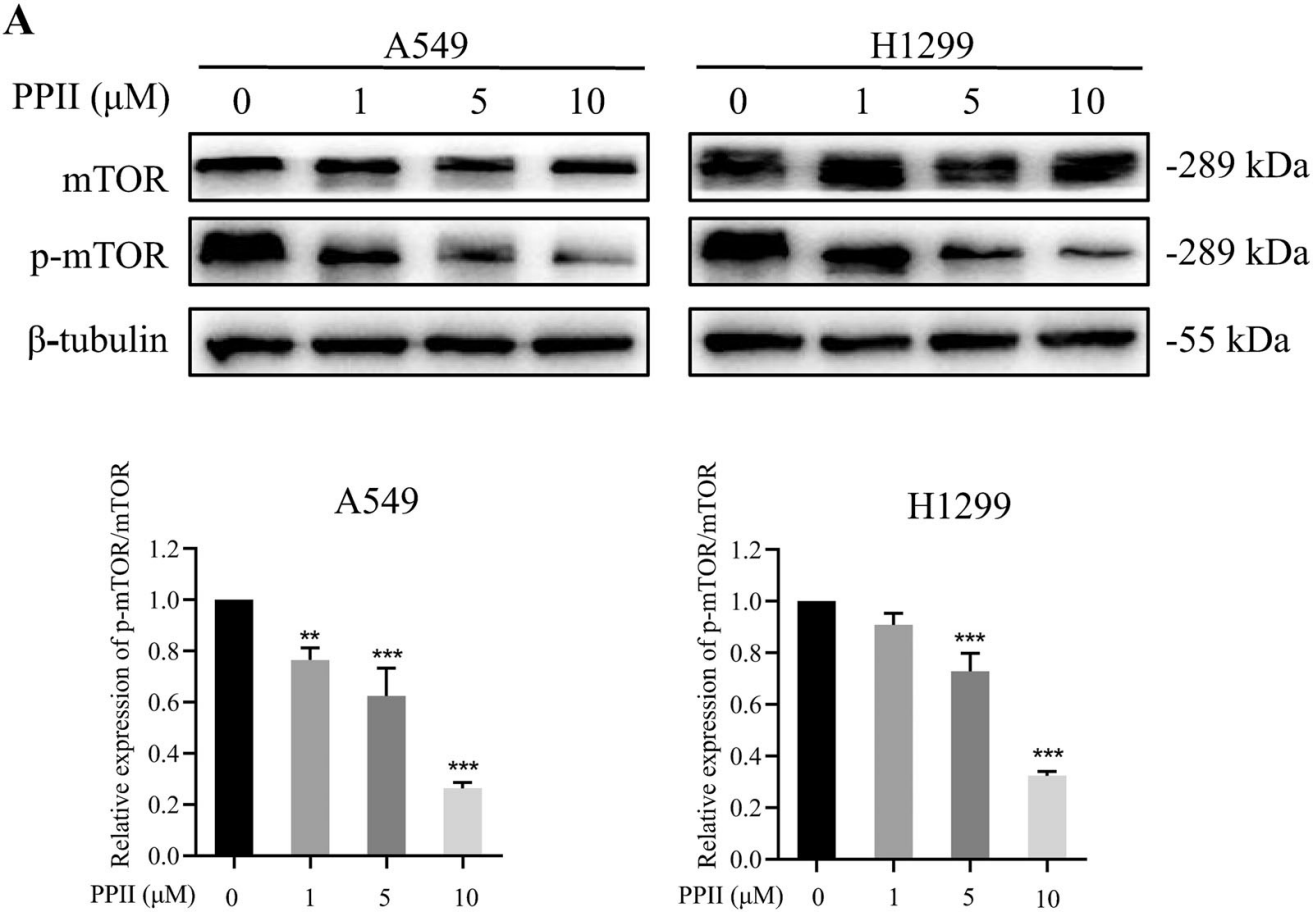
PPII induced autophagy by inhibiting the mTOR pathway in A549 and H1299 cells. Cells were treated with PPII at concentrations of 0, 1, 5, and 10 μM for 24 h. The protein expression of mTOR and p-mTOR was detected by western blotting. Data are mean ± *SD*, representative of three independent experiments. **p < 0.01, ***p < 0.001, compared with the controls.

PPII is known to trigger autophagy in a variety of processes. We investigated the role of PPII on the p-mTOR protein, which play an important role in triggering early cellular autophagy (Kocaturk et al. 2019; Zanini et al. 2020). Therefore, we investigated whether PPII induces autophagy by inhibiting mTOR. According to Figure 5(A), in contrast to the control group, PPII-induced decreased expression of p-mTOR protein, a central molecule of autophagy in A549 and H1299 cells.

### Inhibition of autophagy enhanced the cytotoxicity of PPII to A549 and H1299 cells

**Figure 6.**
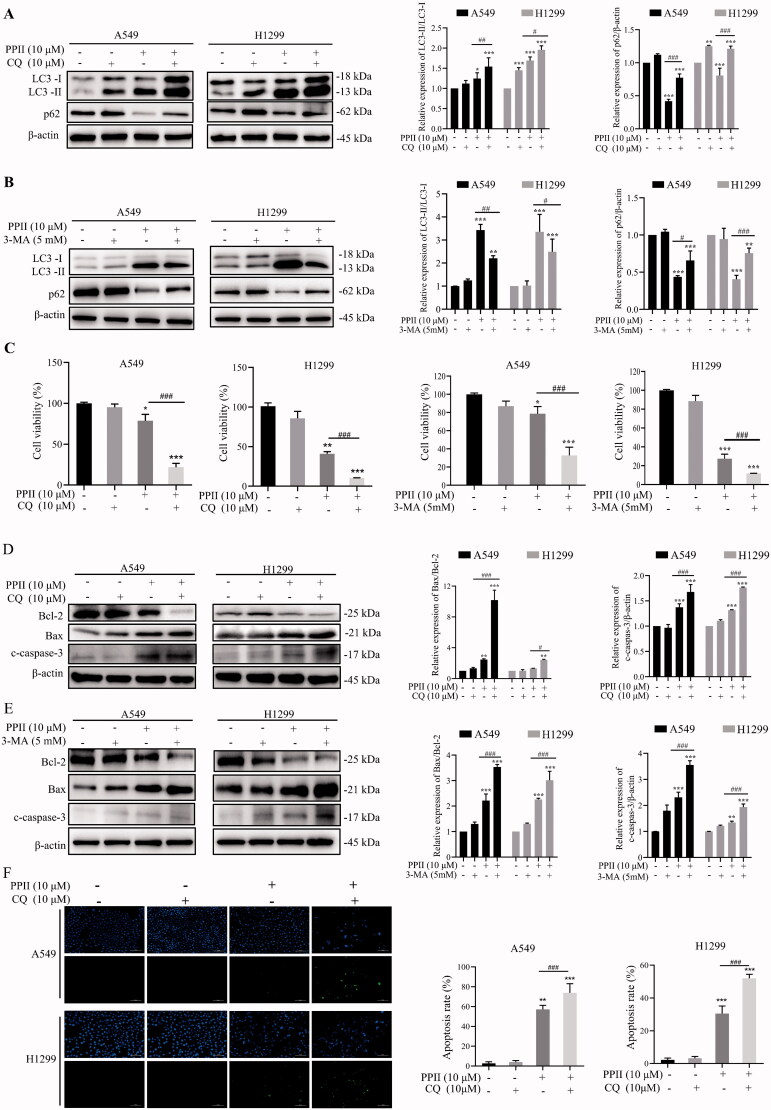
Inhibition of autophagy enhanced the cytotoxicity of PPII to A549 and H1299 cells. A549 and H1299 cells were treated with CQ (10 μM) or 3-MA (5 mM), with or without PPII (10 μM) for 24 h, untreated group was used as control. (A, B, D, E) The protein expression of LC3-II/LC3-I, p62, Bax/Bcl-2, and c-caspase-3 was detected by western blotting. β-Tubulin was used as a loading control. (C) The cell viability was determined by CCK8 assay. (F) The apoptosis was evaluated by TUNEL staining. The corresponding expression levels are shown as bar graphs. Data shown are mean ± SD of three independent experiments. *p < 0.05, **p < 0.01, ***p < 0.001, versus the controls. **^#^***p* < 0.05, **^##^***p* < 0.01, **^###^***p* < 0.001, versus PPII (10 μM).

To further determine the relationship between PPII-induced autophagy and apoptosis, we observed the effect of PPII on A549 and H1299 cells by blocking autophagy with autophagy inhibitor (CQ or 3-MA). Chloroquine (CQ) acts as a late-stage autophagy inhibitor via blocking autophagosome-lysosome fusion (Sharma et al. 2021). Compared with PPII treatment group, western blot analysis showed that the co-treatment of PPII and CQ further increased the ratio of LC3-II/LC3-I, simultaneously increased protein expression of p62 in A549 and H1299 cells, compared with PPII treatment group (Figure 6(A)). In addition, 3-methyladenine (3-MA), which inhibits autophagosome formation at the beginning (Wang et al. 2022). Our results showed that PPII reduced the ratio of LC3-II/LC3-I, while increased the expression of p62, compared with PPII treatment group (Figure 6(B)). The above results indicate that co-treatment of PPII and autophagy inhibitors affects autophagy, further proving the role of PPII in autophagy. To further determine the relationship between autophagy and apoptosis, we evaluated the effect of co-treatment with PPII and autophagy inhibitors in A549 and H1299 cells. The results showed that the co-treatment of PPII and autophagy inhibitors could significantly enhanced cytotoxicity, which reduced the cell viability of A549 and H1299 cells (Figure 6(C)). The co-treatment with autophagy inhibitors promoted the rate of Bax/Bcl-2, meanwhile significantly increased the expression of apoptosis executive protein c-caspase-3, compared with PPII treatment group (Figure 6(D,E)). The results indicated that autophagy inhibitors enhanced the ability of PPII-induced apoptosis. These results were also demonstrated by the TUNEL staining results (Figure 6(F)). Our data indicated that PPII-induced autophagy, which acts as a pro-survival mechanism. Therefore, the co-treatment of PPII and autophagy inhibitors can lead to the death of A549 and H1299 cells.

## Discussion

Rhizoma Paridis has played a noteworthy role in traditional Chinese medicines. Several studies have confirmed that the main antitumor components of Rhizoma Paridis are steroidal saponins (Li et al. 2013). Many steroidal saponins have been isolated and characterised by previous studies, and preliminary studies of anti-proliferation effects on cancer were presented (Liu et al. 2016). Studies have shown that polyphyllin I, polyphyllin II, polyphyllin VI, polyphyllin VII, polyphyllin G, polyphyllin D have been confirmed to have a solid antitumor effect on a broad spectrum of cancers (Chen et al. 2016; Liu et al. 2020; Xiao 2020; Lai et al. 2021; Zhao et al. 2021; Zhong et al. 2021). PPII has been reported to induce the anti-proliferation and pro-apoptosis effects of NSCLC cells (Niu et al. 2020). Our results showed that PPII could inhibit the proliferation of NSCLC cells and induced apoptosis (Figure 1). These results are consistent with the previous results (Wang et al. 2019).

Autophagy is a dynamic process in which part of the cytoplasm is isolated in autophagosomes and then degraded when fused with lysosomes (Chifenti et al. 2013). It is generally believed that LC3-II, as the key autophagy marker, is essential for the formation of autophagosomes (Kabeya et al. 2000). The p62 is negatively correlated with autophagy activity as an autophagy marker. The protein binds to LC3 directly and is degraded during autophagy (Klionsky et al. 2021). In our research, we studied the transformation of LC3-I to LC3-II, and found that the protein level of LC3-II/LC3-I ratio was increased significantly, while p62 was decreased when cells were exposed to PPII (Figure 3(A)). Overall, PPII treatment increased the formation of autophagosomes and autophagic flux. The formation of autophagic vacuoles was detected by MDC staining (Figure 3(B)), and autophagy flux was detected by the dual fluorescence mRFP-GFP-LC3 system. These results further confirmed that PPII induced autophagy (Figure 4). As is well known, cell signalling pathways are involved in the regulation of autophagy, such as the mTOR signalling pathway, which plays an important role in regulating autophagy of cancer cells. Inhibition of mTOR can trigger autophagy and increase autophagy-related protein levels (Callens et al. 2021). Our results showed that p-mTOR was decreased with increasing concentration of PPII (Figure 5), indicating that PPII may induced autophagy by down-regulating the mTOR signalling pathway.

Recent reports have shown that various natural chemicals show antitumor activity by triggering autophagy and apoptosis (Kim et al. 2013, 2014), which shown that there was a close interaction between autophagy and apoptosis. The mode of interaction necessarily involves related proteins such as p53, BH3-only protein, DAPK (death-associated protein kinase), JNK, etc. (Mariño et al. 2014). There are three types of interaction. Firstly, cooperative relationship, autophagy and apoptosis share the goal of promoting cell death (Cao et al. 2014). Secondly, antagonistic relationship, autophagy does not lead to cell death, but rather promotes cell survival. Mitochondrial autophagy is one of them, which can prevent apoptosis by decreasing mitochondrial outer membrane permeability (MOMP) and reducing the release of mitochondrial pro-apoptotic proteins such as cytochrome C and SMAC/DIABLO (Mariño et al. 2014). Moreover, promotional relationship, in which autophagy is not directly inducing cell death, but can ensure the smooth progress of apoptosis as an energy provider (Ito et al. 2005). Therefore, we need corresponding autophagy inhibitors (CQ and 3-MA) or inducers to explore the interaction between autophagy and apoptosis. CQ and 3-MA have been reported to enhance the effect of chemotherapeutic drugs on cervical cancer (Cui et al. 2006), prostate cancer (Kumar et al. 2014), colon cancer (Li et al. 2009), and lung cancer (Pan et al. 2013). Thus, the results showed that the co-treatment of PPII and autophagy inhibitor (CQ and 3-MA) could increase cytotoxicity and promote apoptosis by inhibiting autophagy (Figure 6). These results revealed that PPII-induced autophagy may be a mechanism that promotes cell survival, instead of autophagic cell death. The sensitivity of PPII-induced apoptosis increased through inhibiting the related signalling pathways in NSCLC cells. There may be an antagonistic relationship between autophagy and apoptosis, but the specific mechanism still needs to be further explored.

## Conclusions

Our results indicated that PPII has anti-proliferative and pro-apoptotic effects on A549 and H1299 cells and induced autophagy by down-regulating the mTOR signalling pathway. More importantly, inhibition of autophagy may increase PPII-induced apoptosis of NSCLC cell lines. Thus, PPII-induced autophagy may be a mechanism that promotes cell survival, rather than autophagic cell death. These findings highlight the importance of the relationship between autophagy and apoptosis in regulating NSCLC cells, which may lead to breakthroughs in the identification and treatment of NSCLC patients. However, further studies are needed to confirm our findings.
